# Perception of toothache in adults from state capitals and interior cities within the Brazilian geographic regions

**DOI:** 10.1186/1472-6831-13-35

**Published:** 2013-07-31

**Authors:** Maylu Botta Hafner, Juliana Zanatta, Vera Lucia Rasera Zotelli, Marília Jesus Batista, Maria da Luz Rosário Sousa

**Affiliations:** 1 Department of Social Dentistry/Psychology Applied to Dentistry, University of Campinas–UNICAMP, Piracicaba Dental School (FOP), Av. Limeira, 901-Bairro Areião, CEP: 13414-903, Piracicaba, SP, Brazil

**Keywords:** Toothache, Oral health, Epidemiological survey, Behavior

## Abstract

**Background:**

Studies emphasizing toothache in adulthood are scarce in Brazil. A greater understanding of both the prevalence and the self-perception of pain among individuals in this age group (35 to 44 years old) is important, especially considering that this is an economically active population. To describe reports of oral pain and oral pain-related aspects in from Brazilian state capitals and interior cities.

**Methods:**

The sample comprised 9779 adults residing in the state capitals and interior cities from each Brazilian region in the SB Brazil 2010 report, regarding reports of oral pain and their intensity in the last 6 months. The descriptive analysis comparing pain reports between and within the regions and regression analysis of pain related to socioeconomic aspects per region were performed considering α=0.05 difference.

**Results:**

The highest prevalence of pain was found in the Southeast region (p<0.01), and there was also difference between the state capitals and interior cities in the South (p<0.01), where the prevalence was higher in the capitals, and in the Southeast, where the higher prevalence was in the interior cities (p=0.03). The Northern region had lower pain intensity than the Southeast and Midwest. Comparing pain intensity, only the Northeast region showed statistical difference between state capitals and the interior cities for pain intensity, where the interior cities had higher pain intensity than the three state capitals. Regarding dental office visitations, the Southeast capitals have the highest prevalence (100%) compared to the North and South. The toothache impact on daily activities was as follows: eating difficulty (29.8% to 72.7%), uncomfortable teeth brushing (over 50%), and sleep disturbance (above 13%). Between the Brazilian regions the socioeconomic aspects differ in relation to the pain; the exception being the association between pain, dental care and income, which occurred in the 5 regions. Users of public dental care services were more likely to present pain, comparing to private dental services, OR ranging from 1.72 in the Northeast to 2.85 in the Southeast.

**Conclusion:**

The prevalence of pain was higher among Brazilian adults, impacting some of the daily activities. The data also showed many differences in the prevalence and intensity of pain among both the Brazilian regions and the cities within the same region.

## Background

The International Association for the Study of Pain (IASP) defines pain as an unpleasant sensory and emotional experience associated with actual or potential tissue damage [1]. Pain is always subjective, and individuals learn the application of the word pain through their experiences with injuries suffered since birth [2].

The World Health Organization (WHO) recognizes that pain, suffering, psychological constraints, and social deprivation may result from oral diseases; thus, causing damages at both the individual and the collective levels [3].

Toothache is one of the most common reasons for visiting the dentist [4], since it prevents or hinders daily activities, such as work, play, or interpersonal relationships [5,6].

Despite these limitations caused by pain from dental origin, it takes people, on average, 60 days to consult a professional after the onset of a toothache [7]; thus, resulting in extractions in more than half of the cases [8].

Studies emphasizing toothache in adulthood are scarce in Brazil. A greater understanding of both the prevalence and the self-perception of pain among individuals in this age group (35 to 44 years old) is important, especially considering that this is an economically active population. More precise information on these epidemiological data would allow better targeting of resources for the employment of prevention policies in public health, which could contribute not only to improve the quality of oral health, but also to reduce absenteeism, since tooth pain is the third cause of work absenteeism [9].

Therefore, this paper aims to describe and compare reports of pain and pain-related aspects in adults (35 to 44 years of age) who live in Brazilian state capitals and interior cities located in each of the 5 geographic regions (North, Northeast, Southeast, Midwest, and South).

## Methods

The sample comprised 9779 adults aged 35 to 44 years, of both sexes, being 7333 individuals from state capitals, and the remaining from interior cities. This age group was established as default, by the WHO, for assessing oral health conditions in adults [10].

The data used in the study were collected by trained teams in 27 capital and interior cities from every Brazilian state. Their training consisted of 16 hours of theory, and 20 hours of practice in oral health variables used in the Oral Health Epidemiological Survey-SB Brazil 2010 [11]. Examiners only collect data after obtaining an acceptable degree of uniformity in the procedures, according to the standards set by the WHO [12].

The teams used Personal Digital Assistants (PDAs) for data recording. The data collectors visited randomly selected households, using toothache and oral health care questionnaires.

Each participant signed an informed consent form. This project was approved by the Brazilian National Commission on Research Ethics (CONEP), registration no. 15498.

The data were obtained from the SB Brazil 2010 data bank, which collected them in Brazilian state capitals, divided by regional groups (North, Northeast, Midwest, Southeast, and South), and interior cities from each state. The reports of pain and related aspects were investigated in each region.

This study focused on the pain and its intensity for the last 6 months. For the "reporting pain" variable, the possible answers were: "Yes", "no" or "do not know". For the "pain intensity" variable, the answers were arranged in a numerical scale from 1 to 5, with 1 for the least pain, and 5 for the highest self-reported pain. For this last variable, subjects who answered "no" or "do not know" were excluded.

The variables analyzed were: use of dental services (dental appointments, toothache consultation, and type of treatment), and pain interference with daily activities (pain while eating or drinking, discomfort when brushing, sleeping disturbance, work and/or study affected by the toothache).

These variables were recorded and allocated into categories. The dental appointment variable was dichotomized as “yes” and “no”; the use of dental services as “public” and “private”; reason for dental appointment as “pain” and “others”. The variables: difficulty eating, uncomfortable brushing, and sleep disturbance caused by teeth discomforts were dichotomized into “yes” or “no”.

The demographic and socioeconomic variables in this study were gender (male and female), income (under R$ 250, between R$ 250 and R$ 750 and above R$ 750), education (8 years, 8 years or more), and residents per room (less than 2 residents per room, 2 residents per room or more).

A descriptive analysis was initially performed with separate datafrom the state capitals and other cities. Descriptive statistics, chi square, one-way ANOVA, and the Tukey test were used to assess differences between pain prevalence and its intensity in the Brazilian regions (α=0.05). Binary logistic regression analysis was performed for each macro region of Brazil, with the outcome being pain (pain either reported or unreported in the past 6 months), in addition to the independent variables: gender, geographic location (interior cities or capitals), income, education, residents per room, and type of dental service. Variables with p<0.2 were included in the regression model, considering the p<0.05 significance level.

## Results

Table 1 shows the prevalence of pain in the last 6 months, the average pain intensity in Brazilian adults, and a comparison between the capitals and the interior cities, divided into the North, Northeast, Midwest, Southeast, and South regions.

**Table 1:** Comparison of prevalence and pain intensity in the last 6 months of Brazilian regions

		**Percentage pain 6 months (N)**	Capital versus interior p-value*	Prevalence per region**	**Intensity average**	Capital versus interior p-value*	Intensity average per region**
**North**	Belém	25.60% (127)	0.05	22.63%^A^	3.30	0.38	3.25^A^
	Boa Vista	31.10% (57)			3.45		
	Macapá	24.80% (85)			3.25		
	Manaus	15.70% (36)			3.27		
	Palmas	18.80% (59)			3.01		
	Porto Velho	20.80% (67)			3.38		
	Rio Branco	19.40% (41)			2.89		
	**Inner North**	26.77% (125)			3.48		
**Northeast**	Aracaju	20.10% (43)	0.21	24.16%^A^	2.81^A^	0.00	3.28^AB^
	Fortaleza	25.70% (95)			3.42^AB^		
	João Pessoa	21.70% (46)			3.43^AB^		
	Maceió	24.20% (45)			3.31^AB^		
	Natal	26.90% (47)			2.97^A^		
	Recife	31.50% (45)			3.51^AB^		
	Salvador	24.50% (67)			3.58^AB^		
	São Luis	13.40% (21)			3.14^AB^		
	Teresina	27.50% (75)			3.09^A^		
	**Inner Northeast**	27.00% (115)			3.88^B^		
**Southeast**	Belo Horizonte	27.00% (70)	0.03	27.87^B^	3.35	0.61	3.55^B^
	Rio de Janeiro	18.50% (60)			3.45		
	São Paulo	36.00% (132)			3.67		
	Vitória	29.70% (46)			3.67		
	**Inner Southeast**	32.99% (161)			3.60		
**South**	Curitiba	22.10% (92)	0.00	25.73%^A^	3.20	0.31	3.38^AB^
	Florianópolis	28.30% (62)			3.50		
	Porto Alegre	28.00% (120)			3.46		
	**Inner South**	18.05% (102)			3.15		
**Midwest**	Brasília	23.20% (52)	0.27	21.09%^A^	3.65	0.17	3.58^B^
	Campo Grande	18.00% (68)			3.67		
	Cuiabá	17.60% (28)			3.03		
	Goiânia	26.00% (65)			3.67		
	**Inner Midwest**	23.60% (112)			3.38		
**Brazil**		21.00% (2367)		0.00*	3.20		0.00**

**Legend:** (*) P-value obtained by Chi-square test; (**) P-value obtained by Tukey test; The superscript letters (A and B) indicates that the same letters show no statistical differences and different letters show differences (α<0.05).

The prevalence of toothache in Brazil reached more than a quarter of the population (21%), with a pain intensity of 3.2 (0 to 5). The highest prevalence of pain (over 30% of the population) was found not only in the Southeast cities, but also in the state capitals of Boa Vista, Recife, and São Paulo. These results were inconsistent with São Luis, where the prevalence of pain reached less than 15% of the population. The capitals with the highest prevalence of pain also had the highest pain intensity.

The higher prevalence of pain was found in the Southeast, differing statistically from the pain found in other regions (p<0.01). The intra region analyses found difference in prevalence between the capitals and the interior cities from the South and Southeast regions, but in different ways. In the South, the capitals presented higher prevalence of pain.

Regarding pain intensity, the capitals: Rio Branco (North), Aracaju, and Natal (Northeast) have the lowest averages (less than 3.0), although their percentages of pain complaints in the last six months were not the lowest.

There was a statistical difference for pain intensity among Brazilian regions (p<0.01). It was possible to observe that the reported pain intensity in the Northern region (3.25) was statistically lower than in the Southeast (3.5) and Midwest (3.58) regions, where there were reports of higher intensity.

There were also differences in pain intensity in the analysis between capital and interior cities in the Northeast region (p<0.01). The pain intensity in the interior cities was statistically higher than in the capitals of Teresina (3.09), Natal (2.97), and Aracaju (2.81).

Table 2 shows the aspects related to toothache in the adult population who complained of toothache in the six months prior to the survey. These aspects relate to the search of dental services, and the possible consequences of toothache in daily activities.

**Table 2:** Frequency of aspects related to pain in adults from Brazilian regions (last 6 months)

		**Went to the dentist % (n)**	**Public dental service % (n)**	**Reason–pain % (n)**	**Difficulty in eating% (n)**	**Brushing discomfort % (n)**	**Sleep disturbance % (n)**
**North**	Belém	97.60% (122)	49.20% (60)	25.40% (31)	56.70% (72)	43.30% (55)	34.90% (44)
	Boa Vista	98.20% (56)	60.70% (34)	41.10% (23)	53.60% (30)	49.10% (28)	48.10% (26)
	Macapá	85.90% (73)	68.50% (50)	23.30% (17)	63.50% (54)	40.00% 34)	48.20% (41)
	Manaus	100.00% (36)	60.00% (21)	25.70% (9)	40.00% (14)	37.10% (13)	33.30% (12)
	Palmas	96.60% (56)	50.00% (28)	33.90% (19)	57.60% 934)	47.50% (28)	20.30% (12)
	Porto Velho	98.50% (66)	53.00% (35)	30.30% (20)	58.20% (39)	46.30% (31)	17.90% (12)
	Rio Branco	95.10% (39)	64.10% (25)	13.20% (5)	47.50% (19)	48.80% (20)	19.50% (8)
	Inner North	90.40% (113)	64.50% (71)	35.10% (39)	49.60% (61)	44.40% (55)	35.20% (43)
**Northeast**	Aracaju	100.00% (43)	69.80% (30)	18.60% (8)	57.10% (24)	37.20% (16)	16.30% (7)
	Fortaleza	97.90% (92)	44.60% (41)	35.90% (33)	62.10% (59)	52.10% 949)	35.80% (34)
	João Pessoa	91.30% (42)	66.70% (28)	33.30% (14)	39.10% (18)	37.00% (17)	41.30% (19)
	Maceió	100.00% (45)	34.10% (15)	13.30% (6)	59.10% (26)	47.70% (21)	48.90% (22)
	Natal	83.00% (39)	53.80% (21)	46.20% (18)	29.80% (14)	41.30% (19)	14.90% (7)
	Recife	100.00% (45)	51.10% (23)	35.60% (16)	60.00% (27)	46.70% (21)	44.40% (20)
	Salvador	92.50% (62)	26.70% (16)	27.90% (17)	40.30% (27)	28.80% (19)	13.40% (9)
	São Luis	81.00% (17)	29.40% (5)	23.50% (4)	66.70% (14)	61.90% (13)	35.00% (7)
	Teresina	93.30% (70)	78.60% (55)	28.60% (20)	50.00% (37)	32.00% (24)	40.00% (30)
	Inner Northeast	91.70% (105)	64.80% (68)	28.80% (30)	67.80% (78)	48.70% (55)	43.00% (49)
**Southeast**	Belo Horizonte	100.00% (70)	31.40% (22)	40.00% (28)	63.80% (44)	60.00% (42)	30.00% (21)
	Rio de Janeiro	100.00% (60)	41.70% (25)	26.70% (16)	61.00% (36)	45.00% (27)	27.10% (16)
	São Paulo	100.00% (131)	42.70% (56)	42.00% (55)	72.70% (96)	53.80% (71)	57.60% (76)
	Vitória	100.00% (46)	50.00% (23)	39.10% (18)	54.30% (25)	40.90% (18)	28.30% (13)
	Inner Southeast	94.40% (152)	49.30% (75)	26.30% (40)	53.30% (90)	52.50% (84)	44.10% (71)
**South**	Curitiba	91.30% (84)	52.40% (44)	38.10% (32)	51.10% (47)	37.00% (34)	29.30% (27)
	Florianópolis	100.00% (62)	53.20% (33)	54.80% (34)	38.70% (24)	46.80% (29)	30.60% (19)
	Porto Alegre	98.30% (118)	48.30% (57)	33.90% (40)	65.00% (78)	47.90% (57)	52.50% (63)
	Inner South	94.10% (95)	49.50% (47)	32.60% (31)	57.80% (59)	40.20% (41)	32.40% (33)
**Midwest**	Brasília	94.20% (49)	32.70% (16)	32.70% (16)	69.20% (36)	50.00% (26)	51.90% (27)
	Campo Grande	98.50% (67)	62.70% (42)	52.20% (35)	61.80% (42)	41.20% (28)	38.20% (26)
	Cuiabá	92.90% (26)	38.50% (10)	30.80% (8)	46.40% (13)	39.30% (11)	25.00% (7)
	Goiânia	92.30% (60)	30.00% (18)	43.30% (26)	53.80% (35)	32.30% (21)	37.50% (24)
	Inner Midwest	88.40% (99)	60.60% (60)	30.30% (30)	60.40% (67)	47.30% (52)	45.50% (51)

Regarding "dental appointment", the Southeast capitals had the highest percentages (100%) compared to the North, Northeast, and South capitals. The North and South capitals had the lowest demand for dental appointment.

Concerning "use of public dental services", it was noteworthy that in 13 Brazilian capitals, the demand for these services was above 50%, most of them in the North and Northeast regions. In the interior cities of the Southeast and South regions, the demand for such services was less than 50%.

For "pain as reason for the visit" the percentage ranged from 13.2% (Rio Branco) to 54.8% (Florianópolis). The interior cities from the North, South, and Midwest regions presented the highest values (above 30%).

Among the variables related to toothache which impacted daily activities (eating difficulty, uncomfortable teeth brushing, and sleep disturbance) the difficulty in eating had the highest percentages, ranging from 29.8% (Natal) to 72.7% (São Paulo). Regarding the "uncomfortable teeth brushing" we observed that in most cities (including the interior cities) fewer than 50% of adults who reported pain had this difficulty. For the “sleep disturbance” variable the values were above 13% in all municipalities, reaching the percentage of 57.6% in São Paulo (capital).

With regard to the socioeconomic status and the presence of pain, we observe in Table 3 that all regions presented an association between pain, income, and type of service in all regions. Those who had lower income had a higher prevalence of pain, with the OR varying from 1.69 (Northeast) to 2.88 (Southeast). Those who used public dental care were more likely to present pain, comparing to users of private dental services, with the OR ranging from 1.72 in the Northeast to 2.85 in the Southeast. The geographical location, either capital or interior cities, presented different directions in two regions. In the Southeast there was a higher prevalence of pain in the capitals while in the Northeast, the higher prevalence of pain was in the interior cities.

**Table 3:** Demographic and socioeconomic aspects related to pain in adults from Brazilian regions (last 6 months), Brazil, 2010

	**Variables**		**No pain n %**	**Pain n %**	**OR crude**	**95% CI**	**p**	**OR adjusted**	**95% CI**	**p**
**North**	Dental service	Public	739 (42.6)	324 (58.2)	1.87	1.54-2.27	<0.01	1.52	1.23-1.87	<0.01
		Private	994 (57.4)	233 (41.8)	1			1		
	Education	Up to 8 years	771 (39.7)	303 (51.7)	1.63	1.35-1.96	<0.01	1.28	1.04-1.57	0.02
		> 8 years	1173 (60.3)	283 (48.3)	1			1		
	Income	Up to 250	274 (14.3)	123 (20.8)	2.37	1.78-3.16	<0.01	1.86	1.34-2.58	<0.01
		250-750	985 (51.3)	343 (58.0)	1.84	1.47-2.31	<0.01	1.54	1.20-1.98	<0.01
		More than 750	661 (34.4)	125 (21.2)	1			1		
	Sex	Female	1228 (62.8)	399 (66.8)	1.2	0.98-1.45	0.07			
		Male	728 (37.2)	198 (33.2)	1					
	Resident per room	2 or more	608 (31.1)	222 (37.2)	1.31	1.08-1.59	<0.01			
		Up to 2	1347 (68.9)	375 (62.8)						
	Location	Interior	342 (17.5)	125 (20.9)	1.25	0.99-1.57	0.05			
		Capital	1614 (82.5)	472 (79.1)						
**Northeast**	Dental service	Public	321 (31.2)	201 (43.8)	1.72	1.38-2.16	<0.01	1.4	1.10-1.79	<0.01
		Private	709 (68.8)	258 (56.2)	1			1		
	Education	Up to 8 years	553 (49.4)	280 (60.1)	1.54	1.24-1.92	<0.01	1.31	1.02-1.67	0.03
		> 8 years	567 (50.6)	186 (39.9)	1			1		
	Income	Up to 250	125 (11.4)	72 (15.8)	1.57	1.22-2.02	<0.01	1.87	1.25-2.80	<0.01
		250-750	576 (52.7)	267 (58.7)	1.95	1.36-2.79	<0.01	1.32	1.01-1.74	0.04
		More than 750	393 (35.9)	116 (25.5)	1			1		
	Sex	Female	710 (63.2)	344 (73.3)	1.61	1.61-2.04	<0.01	1.54	1.20-1.98	<0.01
		Male	414 (36.8)	125 (26.7)	1			1		
	Resident per room	2 or more	447 (39.8)	227 (48.4)	1.42	1.14-1.77	<0.01			
		Up to 2	677 (60.2)	242 (51.6)	1					
	Location	Interior	327 (29.1)	161 (34.3)	1.27	1.01-1.06	0.03	1.34	1.04-1.72	0.02
		Capital	797 (70.9)	308 (65.7)						
**Southeast**	Dental service	Public	317 (26.3)	181 (50.4)	2.85	1.24-3.64	<0.01	2.5	1.93-3.26	<0.01
		Private	889 (73.7)	178 (49.6)	1					
	Education	Up to 8 years	475 (38.0)	190 (50.5)	1.67	1.32-2.10	<0.01			
		> 8 years	775 (62.0)	186 (49.5)	1					
	Income	Up to 250	58 (4.7)	42 (11.4)	3.7	2.39-5.72	<0.01	2.88	1.79-4.65	<0.01
		250-750	469 (37.9)	188 (50.9)	2.05	1.60-2.62	<0.01	1.68	1.28-2.20	<0.01
		More than 750	709 (57.4)	139 (37.7)	1			1		
	Sex	Female	803 (64.0)	263 (69.9)	1.31	1.02-1.68	0.03			
		Male	451 (36.0)	113 (30.1)	1					
	Resident per room	2 or more	287 (22.9)	119 (31.6)	1.56	1.21-2.01	<0.01			
		Up to 2	967 (77.1)	257 (68.4)	1					
	Location	Interior	463 (36.9)	102 (27.1)	0.64	0.49-0.82	<0.01	0.51	0.39-0.67	<0.01
		Capital	791 (63.1)	274 (72.9)				1		
**South**	Dental service	Public	373 (34.4)	146 (48.5)	1.8	1.39-2.31	<0.01	1.61	1.23-2.11	<0.01
		Private	712 (65.6)	155 (51.5)	1			1		
	Education	Up to 8 years	562 (48.8)	196 (60.9)	1.63	1.27-2.10	<0.01			
		> 8 years	590 (51.2)	126 (39.1)	1					
	Income	Up to 250	128 (11.4)	65 (20.6)	2.61	1.78-3.82	<0.01	2.29	1.51-3.48	<0.01
		250-750	581 (51.6)	170 (53.8)	1.5	1.12-2.02	<0.01	1.36	1.00-1.85	0.04
		More than 750	416 (37.0)	81 (25.6)	1			1		
	Sex	Female	757 (65.3)	227 (69.8)	1.23	0.95-1.61	0.12			
		Male	403 (34.7)	98 (30.2)	1					
	Resident per room	2 or more	394 (34.0)	134 (41.2)	1.36	1.06-1.76	0.01			
		Up to 2	766 (66.0)	191 (58.8)	1					
	Location	Interior	363 (31.3)	112 (34.5)	1.15	0.89-1.50	0.27			
		Capital	797 (68.7)	213 (65.5)	1					
**Midwest**	Dental service	Public	648 (39.4)	302 (54.2)	1.82	1.50-2.21	<0.01	1.33	1.08-1.65	<0.01
		Private	995 (60.6)	255 (45.8)	1			1		
	Education	Up to 8 years	676 (37.1)	328 (55.1)	2.08	1.73-2.51	<0.01	1.59	1.28-1.97	<0.01
		> 8 years	1145 (62.9)	267 (44.9)						
	Income	Up to 250	344 (19.3)	181 (30.8)	2.91	2.21-3.83	<0.01	2.12	1.55-2.91	<0.01
		250-750	860 (48.3)	302 (51.4)	1.94	1.52-2.48	<0.01	1.65	1.26-2.16	<0.01
		More than 750	575 (32.3)	104 (17.7)	1			1		
	Sex	Female	1176 (64.3)	437 (73.0)	1.5	1.22-1.84	<0.01	1.5	1.20-1.87	<0.01
		Male	654 (35.7)	162 (27.0)	1			1		
	Resident per room	2 or more	666 (36.4)	247 (41.2)	1.23	1.02-1.48	0.03			
		Up to 2	1164 (63.6)	352 (58.8)	1					
	Location	Interior	311 (17.0)	115 (19.2)	1.16	0.92-1.47	0.21			
		Capital	1519 (83.0)	484 (80.8)	1					

## Discussion

The prevalence of pain in the last six months among Brazilian adults can reach up to one third of the adult population in some capitals and/or interior cities, with a mean of pain intensity of approximately 3.2, characterized as a reference between "severe pain" and "very severe pain", according to Silva and Ribeiro-Filho [1]. Pain causes suffering and this study improves the knowledge about this condition, which impacts the active age population; thus, resulting in losses of productivity and decrease in the quality of life.

Despite the high intensity and prevalence of pain in the whole country, the analyses show uneven results between the neighboring regions. The South and Southeast regions have different behaviors in relation to the prevalence of pain, since the highest prevalence of pain in the Southern region is in the capitals, while in the Southeast it is in the interior cities. Perhaps the features of these regions are different or otherwise distributed.

Regarding pain intensity, there are differences between capitals and interior cities in the Northeast region. The interior cities show the highest intensity of pain, statistically different than three capitals. Once more it is possible to justify this difference by the distribution and access to dental resources in this region.

Gibilini *et al.,*[13] surveyed 1612 adults in an epidemiological survey conducted by the Health Department of the State of São Paulo in conjunction with the School of Public Health, from the University of São Paulo (USP), according to the SB Brazil Project methodology. This study found that in the state of São Paulo (Southeast region) the prevalence of dental pain was 34.1%, which indicates that one-third of this population had episodes of pain. According to our survey with the SB Brazil 2010, the data still show that both in the capital and in the interior cities from the state of São Paulo, the prevalence of pain reached one-third of the adult population.

Moreover, the prevalence of pain is high in the Southeast, which causes numerous problems of economic and social order. In the Southeast region there are more frequent visits to the dentist and disturbances caused by toothache. These data were different than the ones reported in another Brazilian study, which found a higher prevalence of pain in the North and Northeast capitals [14]. The geographical location, either interior cities or capitals, remained significant even after adjustments for demographic and socioeconomic variables in the Southeast and Northeast; however, the likelihood of having pain was higher in the Northeast interior cities, and in the Southeast capitals. The migration of Northeasterners to the Southeast in search of better jobs and life opportunities is a noteworthy phenomenon. This fact demonstrates the heterogeneity among Brazilian regions as well as within the regions, between capitals and interior cities, which may have different characteristics in relation to the access to dental services.

A toothache seems to be disabling for the main function-eating-peaking at 72% of the population in São Paulo (capital). This same city reported the highest occurrence of sleep disturbance caused by toothache (57.6%), which can compromise the quality of life of individuals.

For the suffering and limitations it causes, the pain has a major impact in the daily life; thus, having a dramatic effect on society due to the high cost of treatment, and the loss in productivity [15].

In a cross-sectional study with 276 adults from Porto Alegre (South region) in 2005, Gomes and Abegg (2007) [16] described that 73.6% of those reported at least one daily activity affected by dental problems in the past six months. The most affected performance was also related to the eating function (48.6%), followed by a difficulty in performing oral hygiene (38.4%). In this study, the data showed that in this same city, the difficulties in eating and teeth brushing had the highest percentages (65% and 47.9%, respectively).

The demand for public dental services was the highest in the North, and the Northeast regions. This same distribution has been described by Pinto *et al.,*[17] when analyzing the data from the SB Brazil 2003 project. They highlighted the factors associated with the demand for public dental services by the Brazilian adult population, which indicate that in the above mentioned areas the percentage of demand for public dental services was above 60%. The results suggest the probability that in these regions, the public service is the main form of dental care available to the population. These regions have a large low-income population, who depend on public health services. Therefore, people seek the services only on an emergency basis, in the presence of pain.

The type of dental services was associated with the presence of pain in the five Brazilian regions, and the users of public services had a higher prevalence of pain. It is noteworthy that in the Southeast, the likelihood of pain was more prevalent, and the use of public services was higher, compared to the private sector. Lacerda et al. [4] observed a higher prevalence of pain among adults who did not use the dental services provided by their employers; however, according to Bastos *et al.,*[18] the influence of health services needs further clarification, since their attitude may be affected by the social environment, such as home, school, and work.

The perception of pain can be associated with demographic and socioeconomic characteristics as demonstrated in the present study, and others [4,18]. In the present study, women had a higher prevalence of pain in the Northeast and Midwest, but according to Peres *et al.,*[14] the dental pain was higher among Brazilian men. With respect to income and education as socioeconomic factors, there was an association between pain and income in all Brazilian regions and in some regions such as the North, Northeast and the Midwest, education levels was also associated with pain, according to Lacerda *et al.,*[4].

The association of socioeconomic factors with pain highlights the role of social inequalities in health care, since dental pain is strongly associated with oral diseases, mainly the decay [18]. Income and education are determinants of health, and it is proven that individuals who find themselves in situations of socioeconomic disadvantages are at a higher risk of developing oral diseases [19,20], and to suffer dental pain because of these diseases.

The higher prevalence of pain and its intensity across the country results from a curative health policy, which aims at solving the problems instead of preventing them. Thus, future studies should address the factors directly linked to the subjective aspect of pain, as well as seeking ways to minimize their impact on the lives of individuals. This high prevalence and intensity of reported pain in adults indicate the need to encourage awareness and/or perception of dental problems at an earlier stage, favoring a preventive approach instead of the emergency curative, now present in this segment of the population.

## Conclusions

The prevalence and intensity of pain were higher among Brazilian adults, both in capitals and interior cities, as well as the impact on some activities performed by these individuals. The differences found between regions and between capitals and interior cities are many, showing an unequal country regarding health care. New public policies should be directed to prevent episodes of dental pain in all Brazilian regions.

## Competing interests

The authors declare that they have no competing interests.

## Authors' contributions

MBH performed the statistical analysis and drafted the manuscript. JZ participated in the design, and in drafting the manuscript. VLRZ participated in the design of the study. MJB performed the statistical analysis. MLRS participated in its design and coordination, and helped to draft the manuscript. The final manuscript was read and approved by the authors.

## Pre-publication history

The pre-publication history for this paper can be accessed here:

http://www.biomedcentral.com/1472-6831/13/35/prepub
